# Gender Self-Identification: Opinions About Transgender Women from a National Online Survey in Taiwan

**DOI:** 10.1007/s10508-024-02819-3

**Published:** 2024-03-18

**Authors:** Kuo-Yu Chao, Chih-Chiang Chou, Ching-I Chen, Wei Cheng

**Affiliations:** 1 Department of Nursing, Chang Gung University of Science and Technology, Taoyuan, Taiwan; 2 Division of Colon and Rectal Surgery, Chang Gung Memorial Hospital, Linkou, Taiwan; 3 Department of Psychiatry, Centro Hospitalar Conde de São Januário, Sé, Macau SAR, China; 4 Department of Psychiatry, Keelung Hospital, Ministry of Health and Welfare, Kee-Lung, Taiwan; 5 Department of Pathology, Kee-Lung Hospital, Ministry of Health and Welfare, No. 268, Xin 2nd Road Xinyi District, Keelung City, 201203 Taiwan, ROC; 6 School of Nursing, National Taipei University of Nursing and Health Sciences, Taipei, Taiwan; 7 Department of Nursing, Deh Yu College of Nursing and Health, Kee-Lung, Taiwan

**Keywords:** Gender self-identification, Trans women, Trans men, Sex-reassignment surgery, Transgender

## Abstract

Gender self-identification (transgender) is not permitted in most Asian countries. In Taiwan, individuals recognized as transgender must meet requirements mandated by the Gender Recognition Act. Currently, lifting the requirement for proof of sex-reassignment surgery is pending. The aim of this study was to survey a large sample of Taiwanese to gain a better understanding of the general population’s attitudes toward gender self-identification. A self-report survey, entitled “Opinions of Gender Self-Identification,” collected demographic information and responses (agree = 1, disagree = 0) to 14 statements about transgender women and women’s safety, personal rights, and the law; one statement discussed rights of transgender men to give birth; total scores ranged from 0 to 14. The online survey was distributed to non-government organizations across Taiwan and the Taiwanese islands and was available between April 16 and 30, 2022. Most of the 10,158 respondents were female (77.4%); ages of respondents ranged from 15 to > 65 years. The mean total score was 0.95 ± 2.27, indicating respondents strongly disagreed with support for transgender females; 91.56% disagreed with all statements. Although there were significant differences in scores between parents and non-parents, and those ≤ 35 years versus ≥ 36 years (*p* < .01), all strongly disagreed with gender self-identification. Given the majority of respondents were females, survey findings should be regarded with caution. Public acceptance of gender self-identification requires support from its residents. Our findings suggest that gender self-identification has not begun to approach even a moderate level of public support among survey respondents.

## Introduction

Gender identity is a person’s internal sense of being male, female, or “something else” (American Psychological Association, 2023) and is distinct from gender expression, which is “the presentation of an individual including physical appearance, clothing choice, and behaviors that express aspects of gender identity” (American Psychological Association, 2015). Traditionally, individuals are considered to be “gender binary,” meaning they are either male or female (Morgenroth et al., 2021). There is a growing acceptance of individuals who identify as transgender, and in the transgender community the choice for gender self-identification is viewed as a right and one that should be legally recognized (Zimman, 2019).

Transgender individuals are those whose gender identity is incongruent with their natal gender. What was previously referred to as “gender identity disorder” has been removed from the *International Classification of Diseases* and is now “gender incongruence” and is discussed under the chapter on sexual health (World Health Organization, 2022a). The World Health Organization (WHO) refers to the term “gender” as the “socially constructed roles, behaviors, activities, and attributes that a given society considers appropriate for men and women.” Compared to the social characteristics of gender, the notion of sex is largely associated with a person's biological characteristics (World Health Organization, 2022b). In this study we applied the definitions of gender, sex, and transgender (trans women or trans men), in line with those used by the WHO, with the aim of investigating opinions about gender self-identification across Taiwan.

In 2019, Taiwan’s Legislative Yuan approved the Same-Sex Marriage Act, which was the first and only law legalizing same-sex marriage in an Asian country (Zhang, 2019), which was a major advance for the rights of the LGBTQ community. However, in most Asian countries obtaining a legal change from one’s sex assigned at birth to one’s self-identified gender is not permitted or is difficult. In Taiwan and a few states in the USA, a change of gender that is congruent with one’s self-identification on legal forms of identification, such as a driver’s license or voter registration form, requires medical proof of gender affirmation procedures, such as sex-reassignment surgery (SRS), and/or psychiatric documentation justifying a name change (Restar et al., 2020). When a decision by a Taiwanese court found in favor of a plaintiff who sought legal recognition of being transgender without proof of SRS in 2021, the Taiwan legislature began to consider ratification of a law that would allow gender self-identification without a surgical procedure. While this law is only under consideration, there has been vigorous discussion in the public surrounding the appropriateness of ratification.

### Gender Self-Identification in Taiwan

In 2013, the Ministry of Health and Welfare of Taiwan acknowledged that gender self-identification is a human right that must be respected (Ministry of Health & Welfare, 2013). In Taiwan a legal change in gender on government-issued identification cards requires a Taiwanese citizen to have been diagnosed as having gender dysphoria by two psychiatrists and to present a letter from a doctor confirming SRS. This requirement was mandated in 2008 by the Gender Recognition Act (Ministry of Interior, 2008).

The gender-change requirements were partially overturned in 2021, when a court ruled in favor of a plaintiff who claimed the requirement of providing surgical evidence of one’s gender identity violated one’s personal right to privacy right. The plaintiff did provide psychiatric evaluations in favor of a change in name and gender. In September 2021, “he” was permitted to change the gender on the ID card to “she” without proof of SRS (Taipei High Administrative Court, 2021). Although the Gender Recognition Act has not been altered to authorize only a psychiatric evaluation for gender self-identification, the court ruling allows individuals aged 18 years or above, to file for legal recognition of their preferred gender if accompanied by medical certificates from two psychiatrists; each decision is made on a case-by-case basis (Taipei High Administrative Court, 2021). Therefore, legally, medical proof of SRS remains a requirement for individuals wishing to legally change their gender.

### Study Aim

An online opinion survey, “Opinions of Gender Self-Identification” (OGSID), developed by an association of parents, women, and adolescents (age > 15 years), was distributed across Taiwan and the Taiwanese islands. Opinions were determined by mean scores from 14 statements (agree = 1 point, disagree = 0 points) about gender self-identification. In addition to assessing survey scores for all respondents, we also compared scores for different groups of residents based on demographics (gender, age, parental status, education) and supporters versus non-supporters of the Same-sex Marriage Act, and teaching children < 18 years about gender identity. We hypothesized that opinions about gender self-identification would differ between groups. Our findings could help develop interventions to improve acceptance of transgender rights across Taiwan.

## Method

### Participants

A total of 10,528 OGSID surveys were submitted online from April 16 to April 30, 2022. However, three surveys were incomplete, 10 respondents were under 15 years of age, and 357 surveys originated from the same IP address, indicating submissions from the same respondents and invalidating these surveys. Thus, 10,158 surveys were analyzed with a sample loss of 3.5%.

### Measures and Procedure

Data were collected from online surveys available between April 16 and April 30, 2022. Residents of Taiwan (aged 15 years and above) were invited to participate in the online survey through a link provided by a national organization of parents, women, and children by non-government agencies in five regions: northern, middle, southern, and eastern Taiwan; and the outlying islands. In this survey, sample size calculation for ± 1% precision levels, a confidence interval of 95%, and *p* = 0.5 was determined to be 9,604 (Israel, 1992). Considering a 5% attrition rate, 10,084 valid surveys were needed.

An association comprised of parents, women, and adolescents (age > 15 years) developed a self-report survey to assess the opinions of the Taiwanese public toward gender self-identification. Statements on the OGSID were guided by relevant reports from various governments, documents, news, social media, and other literature. Because a meta-analysis of studies conducted in Europe, the USA, and Australia reported a prevalence of 6.8 in 100,000 for trans women (birth assigned males) and 2.6 in 100,000 individuals for trans men (birth assigned females) (Arcelus et al., 2015); in addition, the prevalence of trans women is greater and commands higher visibility in the popular press, 13 of 14 statements focused on trans women; only one was about trans men. The survey began with the following statement and definitions: The purpose of this survey is to gain an understanding how residents of Taiwan view issues surrounding gender identity (gender self-identification) with regard to women’s safety, women’s rights, and the law and society. Please refer to the following definitions when responding to the survey statements: “Trans women” are those whose sex assigned at birth (biological status) was male, but who identify and live as women without female sex-reassignment surgery; ^“^Trans men” are those whose sex assigned at birth (biological status) was female, but who identify and live as men without sex-reassignment surgery; and “Transsexual women” are those assigned male sex at birth and received female sex-reassignment surgery.

The survey was divided into three categories based on socially constructed roles, behaviors, activities, and attributes that a given society considers appropriate for women (World Health Organization, 2022b): women’s safety (5 items), women’s rights (5 items), and law and society (4 items). Respondents answered each statement with agree (1 point) or disagree (0 points). For the category of law and society, there was a third choice for items 11–14 of “no-opinion,” which was not scored. The total score for all items of the OGSID survey ranged from 0 to 14; lower scores indicated lower acceptance of gender self-identification.

Because the survey population was to include adolescents aged > 15 years, face validity was assessed by four senior high school students, two boys and two girls, to evaluate the statements for readability and understandability. Each statement was evaluated on a 5-point Likert scale from 1 = difficult to read or understand to 5 = highly readable and understandable. The mean total score for face validity was 4.25, indicating no changes were needed in the wording of the statements. The content validity index (CVI) of the survey items was examined by a panel of five experts: one psychiatrist with Ph.D. degree specializing in the history of gender dysphoria; a Ph.D. researcher with a major in feminism and gender equality; a research with a master’s in biology and 5-years’ experience in the practice of gender equality; a secretary general of the Women's Association with 5-years’ experience in gender equality practice; and a pediatrician with master’s degree certified as an instructor in gender equity education. The CVI was 93.3%, indicating good validity of the items.

Cronbach’s alpha determined internal consistency for the three categories of the survey: Women’s safety was 0.85, women’s right was 0.78, and law and society was 0.81. The Cronbach’s alpha for the total score on the survey was 0.92. Therefore, the survey had good reliability and validity and could serve as a tool for collecting data about opinions of gender self-identification.

The OGSID survey also included a section on demographic information, which included gender, age, parent or non-parent, education (with or without a college degree), and questions about whether they supported same-sex marriage act or not, agreed with teaching self-exploration of “gender identity,” and acceptance or non-acceptance of gender identification when it was inconsistent with their natal sex for individuals < 18 years.

A third section of the survey allowed respondents to provide qualitative feedback about their opinions. This was an open-ended question, asking, “Is there anything you would like to share about gender self-identification?”

Several non-government organizations comprised of parent groups, LGBT groups, teachers, and students throughout Taiwan were notified of the OGSID survey through their social media sites and heads of the organizations provided a link to the site. Responders could fill out the survey only once, which was determined their Internet Protocol Address (IP address) through SurveyCake, which allows for anonymous collection of data. None of the surveys were distributed by mail; therefore, only individuals with Internet access participated.

### Data Analysis

Quantitative data were analyzed using IBM SPSS Statistics, version 22.0 (Armonk, NY). Descriptive statistics were used for demographic data as well as frequencies of responses to the OGSID survey (*n*, %). To further examine the opinions reported, the responses of agree and disagree was assigned a numerical value of 1 and 0, respectively. However, because statements 11–14 in the category of law and society included an option of “no opinion,” this response was not included in the numerical analysis. Therefore, the total score for the 14-items of the OGSID survey ranged from 0 to 14; scores for three categories ranged from 0 to 5 for “women’s safety,” 0–5 for “women’s rights,” and 0–4 for “law and society.” The lower the score, the greater the level of disagreement with the statement. Mean scores and the standard deviation (SD) for the total score and the three categories were used to compare differences between groups of respondents. We assumed the survey data from this large sample were continuous and normally distributed (central limit theorem). Comparisons of mean scores were analyzed with independent t tests. Significance was set at *p* < 0.05 for statistical comparisons. Significant differences between groups in mean scores for items of the OGSID were determined by calculating Cohen’s (1988) *d*. Cohen’s *d* = 0.2 is considered a “small” effect size, 0.5 represents a “medium” effect size, and 0.8 represents a “large” effect size. An effect size ≥ 0.2 (using the absolute value) was considered significant.

## Results

### Demographics

Most of the survey respondents were female (*n* = 7,863, 77.4%). A small number of respondents (*n* = 111, 1.1%) did not check male or female. Age groups with the most respondents were 25–35 years (25.6%) and 46–55 years (22.5%). More than half of respondents were parents (52.5%), nearly one-third supported the Same-sex Marriage Act (30.9%), and 24.9% supported teaching children < 18 years of age about gender identity. Only 10.1% of respondents agreed that minors with gender dysphoria should be treated with puberty blockers. Details of demographics and attitudes toward gender self-identification are shown in Table 1.

**Table 1:** Demographic data of survey respondents

	*N* = 10,158	
Variables	*n*	%
Sex		
Female	7863	77.4%
Male	2184	21.5%
Undisclosed^a^	111	1.1%
Age, years^b^		
15–18	119	1.2%
19–24	794	7.8%
25–35	2598	25.6%
36–45	1886	18.6%
46–55	2284	22.5%
56–65	1695	16.7%
> 65	782	7.7%
Parent		
Yes	5338	52.5%
No	4820	47.5%
College degree		
Yes	8,712	85.8%
No	1,446	14.2%
Support the same-sex marriage act		
Yes	3140	30.9%
No	5492	54.1%
No-opinion	1526	15.0%
Support teaching children < 18 years about self-exploration of “gender identity” and acceptance of inconsistency with natal sex		
Agree	2533	24.9%
Disagree	6421	63.2%
No opinion	1204	11.9%
Use of puberty blockers for minors with gender dysphoria		
Agree	1021	10.1%
Disagree	7766	76.5%
No opinion	1371	13.5%
Place of residence		
Northern Taiwan	6275	61.8%
Middle Taiwan	1419	14.0%
Southern Taiwan	2082	20.5%
Eastern Taiwan	251	2.5%
Living outside the main island of Taiwan	131	1.3%

^a^Although some respondents did not disclose their gender, some respondents in the open-feedback noted being gender neutral, non-binary, asexual, bisexual, transsexual (having received SRS), trans woman, or having gender dysphoria

^b^Respondents were not asked to report their age; they only checked one of the seven age ranges and there was no upper age limit

### Opinions from the OGSID Survey

Most respondents (91.6%) disagreed with all 14 items of the survey, indicating an overwhelming negative attitude about gender self-identification and its impact on women’s safety, women’s rights, and the law and society in Taiwan (Table 2). Only two statements exceeded an agreement of more than 10% of respondents: item 5, regarding allowing trans women to sell women’s underwear and to provide personal services in hair salons and spas (14.4%), and item 14, which suggested trans men should be allowed to become pregnant and give birth to babies (15.4%). Details of responses to the survey statements are shown in Table 2.

**Table 2:** Responses to the survey on Opinions of Gender Self-Identification for individuals in Taiwan (*N* = 10,158)

	Opinion		References
	Agree	Disagree	
Category	*n* (%)	*n* (%)	
**Women’s safety**			
1. Trans women ^a^ can use women’s public toilets	624 (6.1%)	9534 (93.8%)	Newman (2019)
2. Trans women can use spas that serve unclothed women	200 (2.0%)	9058 (98.0%)	Liu (2022)
3. Trans women can live in female student dormitories	378 (3.7%)	9780 (96.3%)	Chen (2022)
4. Trans women can be housed in female jails	458 (4.5%)	9700 (95.5%)	Walker (2023)
5. Trans women can work selling women’s underwear and providing women’s personal services at hair salons and spas^‡^	1462 (14.4%)	8696 (85.6%)	(Helen, 2017; Wang, 2021)
**Women’s rights**			
6. A woman cannot refuse to be serviced by a trans woman in spas, or hair salons^‡^	666 (6.6%)	9492 (93.4%)	(Helen, 2017; Wang, 2021)
7. It is fair for the Olympics to allow trans women to compete in women’s sporting events	427 (4.2%)	9731 (95.8%)	Axon (2021)
8. It is fair the 2023 National High School Games to allow trans girls to compete in girls’ sporting events	336 (3.3%)	9822 (96.7%)	Long (2022)
9. It is appropriate for trans women to hold a reserved political seat for women in the Legislative Yuan of Taiwan	674 (6.6%)	9484 (93.4%)	Shaw (2020)
10. Trans^a^ or transsexual^b^ women have the right to take “menstruation leave” without revealing they are transgender	605 (6%)	9553 (94.0%)	Chen (2022)
**Law and society**			
11. The court ruled that trans women without sex-reassignment surgeries could change their gender to “female” on the ID card in Taiwan. I agree with this decision	638 (6.3%)	9072 (89.3%)	(Taipei High Administrative Court, 2021)
12. The Executive Yuan plans to legislate gender-self-identification without sex-reassignment surgeries (SRS). I agree with this decision	890 (8.8%)	8928 (87.9%)	(Executive Yuan, 2022a)
13. Offences committed by trans women should be recorded as female crime statistics	425 (4.3%)	9356 (92.1%)	(Dodds, 2022)
14. Trans men^d^ who identify as male should be allowed to become pregnant and give birth to babies	1569 (15.4%)	7115 (70.0%)	(Hattenstone, 2019)
Overall opinion (Mean % for all questions ± SD)	6.6% (± 3.9%)	91.6% (± 7.1%)	

*SD* standard deviation

^a^Trans women are those assigned male sex at birth, but who identify and live as women without sex-reassignment surgery

^b^Transsexual women are those assigned male sex at birth and received female sex-reassignment surgery

^c^Responses to questions 11–14 for this category included, “no opinion.” Therefore total is < 100%

^d^Trans men are those assigned female sex at birth, but who identify and live as men without SRS

^‡^These questions were designed to address recent reports in the news: One involved a trans woman with “stubble and a beard” who was providing pap smears for women in England, and the other involved a male employee entering a women’s spa in Taiwan

### Percent of Respondents Who Agreed, Disagreed, and Summed Scores on the OGSID Survey

There was not a large difference in the mean percent of respondents who disagreed with the statements for each category or all 14 statements on the OGSID survey (Table 3). Most respondents disagreed with the statements for the three categories (range = 84.83–94.66%). The summed scores for the items in the three categories and the total for the 14 items further illustrate the low level of agreement with the statements about gender self-identification. The mean summed total score for all 14 items (range = 0–14 points) was 0.95 (SD = 2.27).

**Table 3:** Responses from all respondents (*N* = 10,158) from the survey on Opinions of Gender Self-Identification: Percent who agreed and disagreed and summed scores

Variable	Agree	Disagree	Summed scores^a^	
	Mean % ± SD	Mean % ± SD	Mean ± SD	Range
Total for category				
Women’s safety (5 items)	6.15% ± 4.85%	93.85% ± 4.85%	0.307 ± 0.88	0–5 points
Women’s rights (5 items)	5.34% ± 1.51%	94.66% ± 1.51%	0.267 ± 0.79	0–5 points
Law and Society (4 items)	8.70% ± 4.83%	84.83% ± 10.04%	0.389 ± 0.93	0–4 points
Total (14 items)	6.59% ± 3.92%	91.56% ± 7.13%	0.95 ± 2.27	0–14 points

*SD* standard deviation

^a^Summed scores the items (statements) in each category and summed scores for all items: agree = 1 point; disagree = 0 points

### Differences Between Mean Summed Item Scores and Characteristics of Respondents

We examined if the low level of agreement differed between respondents according to characteristics: female versus male; parents versus non-parents; ≤ 35 years versus ≥ 36 years; college degree (yes vs. no); supporters of same-sex marriage (yes vs. no); and teaching children < 18 years of age about gender identity (yes vs. no). We examined differences for the summed item scores for the three categories (Table 4); *p*-values were considered significant based on the effect size calculated with Cohen’s *d*. For the categories of women’s safety and law and society, there was no difference between respondents with or without a college degree, suggesting education did not impact viewpoints of respondents. Non-parents, those whose age was ≤ 35 years, and supporters of same-sex marriage and teaching about gender identity scored significantly higher (all *p* < 0.01, Cohen’s *d* > 0.20). Scores for all three categories were significantly lower for female respondents compared with males (all *p* < 0.01, though all the absolute values of Cohen’s *d* were not over 0.2). Differences in summed scores between groups for the category of women’s rights only differed for supporters of same-sex marriage and teaching children about gender identity (both *p* < 0.01, Cohen’s *d* > 0.20). However, in all cases where differences were significant, the higher scores remained at a low level of agreement.

**Table 4:** Differences between mean summed item scores for participants’ characteristic on the survey of Opinions of Gender Self-Identification (*N* = 10,158): Categories of women’s safety, women’s rights and law and society

Category/Characteristic	Mean (SD)^a^	*t*	p*	Cohen’s *d*^b^
**Women’s safety (range = 0–5 points)**				
Sex		− 7.01	** < .01**	− 0.19
Female (*n* = 7,822)	0.26 (0.78)			
Male (*n* = 2,166)	0.44 (1.12)			
Parental status		− 11.52	** < .01**	− 0.23^†^
Yes (*n* = 5,306)	0.21 (0.74)			
No (*n* = 4,791)	0.41 (1.01)			
Age		12.75	** < .01**	0.28^†^
≤ 35 years (*n* = 3,511)	0.48 (1.07)			
≥ 36 years (*n* = 6,647)	0.22 (0.75)			
College degree		0.21	0.79	0.01
Yes (*n* = 8,712)	0.31 (0.88)			
No (*n* = 1,446)	0.30 (0.93)			
Supporters of same-sex marriage act		22.95	** < .01**	0.57^†^
Yes (*n* = 3,124)	0.64 (1.27)			
No (*n* = 5,846)	0.10 (0.45)			
Supporters of teaching children < 18 years about self-exploration of gender identity and acceptance of inconsistency with natal sex		27.59	** < .01**	0.76^†^
Yes (*n* = 2,533)	0.89 (1.45)			
No (*n* = 6,421)	0.09 (0.37)			
**Women’s rights (range = 0–5 points)**				
Sex		− 5.97	** < .01**	− 0.16
Female (*n* = 7,822)	0.23 (0.71)			
Male (*n* = 2,166)	0.37 (0.97)			
Parental status		− 0.36	.723	0.0
Yes (*n* = 5,306)	0.26 (0.77)			
No (*n* = 4,791)	0.26 (0.80)			
Age		2.02	**.043**	0.05
≤ 35 years (*n* = 3,511)	0.29 (0.85)			
≥ 36 years (*n* = 6,647)	0.25 (0.75)			
College degree		− 4.14	** < .01**	− 0.13
Yes (*n* = 8,712)	0.25 (0.75)			
No (*n* = 1,446)	0.36 (0.98)			
Supporters of same-sex marriage act		15.69	** < .01**	0.26^†^
Yes (*n* = 3,124)	0.46 (1.09)			
No (*n* = 5,846)	0.14 (0.50)			
Supporters of teaching children < 18 years about self-exploration of gender identity and acceptance of inconsistency with natal sex		20.86	** < .01**	0.57^†^
Yes (*n* = 2,533)	0.65 (1.24)			
No (*n* = 6,421)	0.12 (0.44)			
**Law and society (range = 0–4 points)**				
Sex		− 2.70	** < .01**	** − **0.07
Female (*n* = 7,822)	0.37 (0.88)			
Male (*n* = 2,166)	0.44 (1.05)			
Parental status		− 12.58	** < .01**	− 0.25^†^
Yes (*n* = 5,306)	0.28 (0.82)			
No (*n* = 4,791)	0.51 (1.03)			
Age		14.68	** < .01**	0.32^†^
≤ 35 years (*n* = 3,511)	0.59 (1.07)			
≥ 36 years (*n* = 6,647)	0.28 (0.83)			
College degree		1.44	.062	0.03
Yes (*n* = 8,712)	0.39 (0.93)			
No (*n* = 1,446)	0.36 (0.92)			
Supporters of same-sex marriage act		31.49	** < .01**	0.77^†^
Yes (*n* = 3,124)	0.85 (1.28)			
No (*n* = 5,846)	0.11 (0.44)			
Supporters of teaching children < 18 years about self-exploration of gender identity and acceptance of inconsistency with natal sex		35.60	** < .01**	0.97^†^
Yes (*n* = 2,533)	1.12 (1.41)			
No (*n* = 6,421)	0.11 (0.41)			

*SD* standard deviation

*p < 0.05 values are highlighted in bold

^**a**^Summed scores for each item (statements): agree = 1 point; disagree = 0 points

^b^Cohen’s *d* (absolute value): small (*d* = 0.2), medium (*d* = 0.5), large (*d* = 0.8)

^†^Cohen’s *d* ≥ 0.2

We also examined differences between these groups by summing all item scores (total score) (Table 5) with *p*-values considered significant based on the effect size calculated with Cohen’s *d*. As with the summed item scores, respondents who were non-parents, whose age was ≤ 35 years, were supporters of same-sex marriage and teaching about gender identity had significantly higher total scores (all *p* < 0.01, Cohen’s *d* > 0.20). Females and respondents with college degree had significant lower total scores than their matched pairs (*p* < 0.01, though the absolute values of Cohen’s *d* were not over 0.2). Taken together, although there were differences between respondents based on their characteristics, our findings demonstrated gender self-identification was viewed as having a negative impact on the areas surveyed.

**Table 5:** Differences between mean total scores (all categories) for participants’ characteristic on the survey of Opinions of Gender Self-Identification (*N* = 10,158)

Characteristic	Mean (SD)^a^	*t*	p*	Cohen’s *d*^b^
Sex		− 5.70	** < .01**	− 0.15
Female (*n* = 7,822)	0.86 (2.04)			
Male (*n* = 2,166)	1.23 (2.83)			
Parental status		− 9.79	** < .01**	− 0.20^†^
Yes (*n* = 5,306)	0.74 (1.99)			
No (*n* = 4,791)	1.19 (2.53)			
Age		11.68	** < .01**	0.25^†^
≤ 35 years (*n* = 3,511)	1.34 (2.64)			
≥ 36 years (*n* = 6,647)	0.75 (2.02)			
College degree		− 1.08	** < .01**	− 0.03
Yes (*n* = 8,712)	0.94 (2.21)			
No (*n* = 1,446)	1.02 (2.53)			
Supporters of same-sex marriage act		26.55	** < .01**	0.66^†^
Yes (*n* = 3,124)	1.95 (3.26)			
No (*n* = 5,846)	0.35 (1.11)			
Supporters of teaching children < 18 years about self-exploration of gender identity and acceptance of inconsistency with natal sex		31.73	** < .01**	0.88^†^
Yes (*n* = 2,533)	2.65 (3.66)			
No (*n* = 6,421)	0.31 (0.88)			

*SD* standard deviation

*p < 0.05 values are highlighted in bold

^**a**^Summed scores all 14 items (statements): agree = 1 point; disagree = 0 points; range = 0–14

^b^Cohen’s *d* (absolute value): small (*d* = 0.2), medium (*d* = 0.5), large (*d* = 0.8)

^†^Cohen’s *d* ≥ 0.2

### Personal Feedback About Gender Self-Identification

We collected responses to the open-ended question as anonymous feedback. A total of 2,103 respondents provided the feedback (20.7%), of which 77.6% were females (*n* = 1,631); and 49.7% were parents (*n* = 1,046). Some respondents provided information that they identified as gender neutral, non-binary, asexual, bisexual or transsexual (having received SRS), trans woman, or having gender dysphoria. All responses were read and then grouped and labeled based on similarities of respondents’ concerns, which were categorized as follows: security; women’s rights, judicial hegemony, education; gender binary; and gender diversity. Descriptions of the categories and concerns are presented in Table 6 and summarized below.

**Table 6:** Summary of feedback from the open-ended survey question: Categories, description, and opinions

Category	Description	Opinions
1. Security crises	Gender self-identification abdicates women’s safety	There have been many sexual assaults of women by trans women in areas considered exclusive private spaces for women. (Non-parent female, 46–55-year-old, non-supporter of Same-Sex Marriage Act) Trans women’s penises should not be exposed in single-sex spaces for women (women’s toilets and locker rooms, spas, female dormitories). (Non-parent male, 36–45-year-old, supporter of Same-Sex Marriage Act)
2. Women’s rights	Gender self-identification violates women’s right	Allowing trans women to participate in women’s sporting events or hold a reserved political seat designated for a woman in the Legislative Yuan of Taiwan denies women their rights. (Mother, 25–35-year-old, no opinion about Same-Sex Marriage Act) Trans women should not be permitted to take menstrual leave without menstruation. (Non-parent female, 19–24-year-old, supporter of Same-Sex Marriage Act)
3. Judicial hegemony	Gender self-identification is a means of legal persecution of women in Taiwan	Laws should protect people rather than loosening gender-change standards, which creates confusion. (Non-parent female, 25–35-year-old, supporter of Same-Sex Marriage Act) Gender self-identification forces women to leave the safe zone and oppresses women in Taiwan. (Non-parent female, 25–35-year-old, supporter of Same-Sex Marriage Act)
4. Chaos of education	Gender concept and identification are influenced by Gender self-identification	Gender concept and identification need to be confirmed and clarified before 18 years old; gender self-identification will confuse students. (Mother, 56–65-year-old, no opinion about the Same-Sex Marriage Act) Teaching children about gender self-identification results in gender confusion among minors. (Mother, 25–35-year-old, non-supporter of Same-Sex Marriage Act)
5. Gender binary	Classification of people as males or females	People are born as males or females, and society and education should teach children to know their natal sex. (Father, 36–45-year-old, supporter of Same-Sex Marriage Act) Boys are boys, girls are girls, these are facts and truth. (Mother, 56–65-year-old, non-supporter of Same-Sex Marriage Act)
6. Gender diversity	Breaking stereotypes of males and females	Gender is diversity, trans women should not put themselves into stereotype of gender binary. (Non-parent female, 19–24-year-old, no opinion of the Same-Sex Marriage Act) They say that gender self-identification is breaking stereotypes, but actually, it consolidates stereotypes. (Non-parent female,,36–45-year-old, supporter of Same-Sex Marriage Act) No one should be treated unequally because of their gender identity or gender diversity. (Non-parent male, 25–35-year-old, supporter of Same-Sex Marriage Act)

Comments from males and females as well as supporters and non-supporters of the Same-Sex Marriage Act indicated security was a concern. They commented that the safety of women and children is more important than so-called rights of transgender people, which would be elevated by ratifying gender self-identification. Parents voiced concern that daughters’ rights would be violated by the gender self-identification law. They worried if natal women compete with trans women, who are more powerful and suitable for sports, natal women will have no chance to win the award.

Women objected to the gender self-identification law because they believed it was a form of female oppression, regardless of whether they were parents or non-parents. They said people cannot distinguish the difference between sex offenders and trans women, and the law should protect women rather than loosen the standards for gender change.

Several mothers expressed concern about the influences of gender self-identification on children. They believed teaching such information to minors caused children to be confused about gender and did not promote mental health stability.

Some respondents praised gender diversity, and they wondered why trans women put themselves into the stereotype of gender binary. However, other respondents who also supported gender diversity agreed with all the statements about gender self-identification. They said no one should be treated unequally because of their gender identity or gender diversity.

## Discussion

Transgender individuals face multiple difficulties when one’s name and sex on legal documents do not match their gender identity or expression. The discrimination and stigma of being part of the LGBT community has been associated with high levels of suicide internationally, including Taiwan (Wang et al., 2021). Compared with individuals who identify as lesbian, gay, or bisexual, transgender adults in Taiwan have higher levels of depressive symptoms, internalized stigma, lower level of self-esteem, and less perceived formal and informal social support (Wang et al., 2021). Analysis of our survey data indicated an overwhelming majority of respondents (91.6%) did not support gender self-identification, suggesting ratification by the legislature of Taiwan of a law granting individuals gender self-identification rights without proof of SRS would not be well-received by the respondents.

When responses to items were summed for all three categories and compared based on characteristics of respondents, mean scores were significantly higher for non-parents compared with parents, respondents ≤ 35 years old compared with those old ≥ 36 years, supporters of the same-sex marriage act compared with non-supports, and supporters of teaching children < 18 years about self-exploration of gender identity compared with non-supporters. However, despite the significant differences in scores, none of the higher scoring groups were supporters of gender self-identification. Responses to the open-ended question were also overwhelmingly negative.

One important aspect of our findings was the low level of acceptance of gender self-identification by the survey respondents. This was an unexpected finding given that Taiwan was the first country in Asia to legalize same-sex marriage, has a high proportion of gender-neutral toilets (17.4%) (Executive Yuan, 2022b), and is considered to be the most LGBTQ-friendly nation in Asia (Editorial, 2023). The findings were also inconsistent with gender spectrum ideation education, begun in Taiwan in 2012, teaching that multi-sexes/genders are present in our society, gender identity is more important than biological sex, and “you can choose whatever sex/gender you want to be” (Chao et al., 2023; Kung, 2020). One explanation is that the low opinion scores for the survey categories may not reflect the respondents’ attitudes toward transgender individuals, such as people not understanding transgenders and their needs, which were not included in the survey. Therefore, future surveys should include specific questions that ask the acceptance and supports of transgenders with transgender inclusion.

This low acceptance of gender self-identification has been demonstrated in other countries as well as Taiwan. Gender self-identification policies have resulted in young girls in the UK avoiding school because of fear sharing gender neutral toilets (unisex) with male students, which included menstruating girls remaining at home for fear of feeling shame, refusing to urinate all day or not drinking liquids in school (Manning, 2019). Parents and children in the UK are also concerned about the right of trans women to use women’s toilets, partly due to an 18-year-old trans woman sexually assaulting a 10-year-old girl in a female toilet (Newman, 2019).

Policies permitting gender self-identity have been made without the support of women or parents (Crane, 2021). Therefore, when disruptions occur in the public, the outcry from women is particularly strong. One example was the case of a 37-year-old man who entered a women’s spa using a forged identity card and insisted his gender was female in Taiwan. This incident caused a wave of fear for women throughout Taiwan (Liu, 2022).

Legal gender affirmation is not only about transgenders’ rights but also about the impacts on health outcomes; however, studies reported mixed results (King & Gamarel, 2021). One means of improving health outcomes for transgender individuals is to increase family and informal support (Lerner & Lee, 2021; Wang et al., 2021), reduce exposure to abuse, and provide resources for trauma-informed care (Becerra et al., 2021). Increasing positive attitudes by the general population may have salutary effects on the health of transgender individuals. One systematic literature review of studies on transgender youth indicated several factors were protective against poor outcomes, which included problem-solving skills, supportive educators, transgender role models, helpful service providers, peer support/acceptance, anti-bullying/-harassment policies in schools, social support, and transgender health and social services (Johns et al., 2018). Another systematic review of 14 quantitative studies examined if there were protective factors against self-stigmatization and negative mental health outcomes in trans populations (Inderbinen et al., 2021). They found community connectiveness was a strong protective factor against depression, anxiety, and suicidal tendency. Taiwan’s data provided more information of psychological health in people with gender dysphoria (Chao et al., 2023). Comorbidities of attention-deficit/hyperactivity disorder, autism spectrum disorder, psychosis, and comorbid depression were more likely in adolescents and adults with gender dysphoria in Taiwan (Chao et al., 2023). Therefore, providing educational outreach programs, providing support for transgender men and women through the presence of social support/acceptance, and transgender health and social services could increase resilience (Johns et al., 2018) and protect against self-stigmatization and improve mental health outcomes in trans populations (Inderbinen et al., 2021). Amending Gender Recognition Act should include ways to protect and improve the psychological health of transgender adults.

Governments have the power and a duty to put an end to the ordeal faced by trans and gender-diverse persons and foster their inclusion (United Nations, 2023). Therefore, governments and researchers should conduct further research to understand the source of this lack of acceptance of gender self-identification with initiatives that provide education and an understanding of the trans populations to reduce discrimination and stigma.

### Limitations

There are several limitations. First, although this study fit the statistical assumptions to be continuous and normally distributed by central limit theorem, it still had the possibility that it is not a perfect normal distribution. Second, online surveys cannot accurately describe the population to which the surveys are distributed, and the respondents may be biased (Andrade, 2020). Our online survey data were also limited by a majority of respondents (77%) being female. Although responses from males indicated they shared similar negative opinions about the topic, we cannot exclude the possibility that our sample was biased toward respondents who disapproved of gender self-identification and wished to share their opinions. The attitudes toward transgenders of Taiwanese people may be recognized worse than Western individuals because of more women or lesbians (according to the feedback) answering this survey. More detailed designs about understanding transgenders and their needs, and quantitative study are warranted in the future.

### Conclusion

The findings of this survey demonstrated a lack of support for gender self-identification from respondents, and the potential implications are that most people did not understand transgenders and their needs. Therefore, more communications and discussions with different groups and in public are needed to find the balance between women’s rights and transgenders’ rights.

## Data Availability

The datasets generated and/or analyzed during the current study are available from the corresponding author on reasonable request.

## References

[CR1] American Psychological Association. (2015). Guidelines for psychological practice and transgender and gender nonconforming people. *American Psychologist*, *70*(9), 832-864. 10.1037/a0039906 10.1037/a0039906 26653312

[CR2] American Psychological Association. (2023, June 6). *Understanding transgender people, gender identity and gender expression.* Sexual orientation and gender diversity. https://www.apa.org/topics/lgbtq/transgender-people-gender-identity-gender-expression

[CR3] Andrade C (2020). The limitations of online surveys. Indian Journal of Psychological Medicine.

[CR4] Arcelus J, Bouman WP, Van Den Noortgate W, Claes L, Witcomb G, Fernandez-Aranda F (2015). Systematic review and meta-analysis of prevalence studies in transsexualism. European Psychiatry.

[CR5] Axon, R. (2021, August 2). New Zealand’s Laurel Hubbard makes history as first transgender woman to compete at Olympics. *USA TODAY.* https://www.usatoday.com/story/sports/olympics/2021/08/02/laurel-hubbard-becomes-openly-first-trans-woman-compete-olympics/5451329001/

[CR6] Becerra MB, Rodriquez EJ, Avina RM, Becerra BJ (2021). Experiences of violence and mental health outcomes among Asian American transgender adults in the United States. PLoS ONE.

[CR7] Chao KY, Chou CC, Chen CI, Lee SR, Cheng W (2023). Prevalence and comorbidity of gender dysphoria in Taiwan, 2010–2019. Archives of Sexual Behavior.

[CR9] Chen, C. J. (2022, May 17). 成大跨性別生搬進女宿惹爭議 女學生怒斥:「不要犧牲女性空間去換性別友善」[Transwoman students move into female dormitories of Cheng Kiung university, female students angrily denounced: “Don't sacrifice women's space for gender friendly”]. *Newtalk*. https://newtalk.tw/news/view/2022-05-17/756072

[CR8] Chen, Q. F. (2022, Feb 10). 男跨女跨性別者請生理假?勞動部: 不可以 [Can trans women take “menstruation leave”?]. Ministry of Labor said “No”] *CNA*. https://www.cna.com.tw/news/ahel/202202100186.aspx

[CR10] Cohen J (1988). Statistical power analysis for the behavioral sciences.

[CR11] Crane, E. (2021, October 13). Parents demand Virginia superintendent be fired over alleged sex assault cover-up. *New York Post.* https://nypost.com/2021/10/13/parents-demand-superintendent-be-fired-for-alleged-sex-assault-cover-up/

[CR12] Dodds, I. (2022, April 12 ). Home Office asks police to record trans people using their birth sex in crime statistics. *INDEPENDENT*. https://www.independent.co.uk/news/uk/home-news/home-office-trans-crime-statistics-grc-birth-sex-b2056345.html

[CR13] Executive Yuan. (2022a). 行政院「性別變更要件法制化及立法建議」研究案 [Research of suggestion of legislating gender-self-identification without sex-reassignment surgeries (SRS)]. https://gec.ey.gov.tw/File/641C9A1CEFC38DB0/609f345c-7b49-4f77-a3c4-d2d028f02502?A=C

[CR14] Executive Yuan. (2022b). 國情統計通報 [National Statistical Report]. https://www.dgbas.gov.tw/public/Data/27141616222FZ3FA2L.pdf

[CR15] Editorial. (2023). The LGBTQ movement in Taiwan. *OFTAIWAN*. https://oftaiwan.org/social-movements/lgbtq-movement-in-taiwan/.

[CR16] Hattenstone, S. (2019, April 20). The dad who gave birth: ‘Being pregnant doesn't change me being a trans man’ *The Guardian.* https://www.theguardian.com/society/2019/apr/20/the-dad-who-gave-birth-pregnant-trans-freddy-mcconnell

[CR17] Helen, N. (2017, December 31). The female NHS nurse I asked for came with stubble. *The Times* https://www.thetimes.co.uk/article/the-female-nhs-nurse-i-asked-for-came-with-stubble-83rq9p0gg

[CR18] Inderbinen M, Schaefer K, Schneeberger A, Gaab J, Garcia Nuñez D (2021). Relationship of internalized transnegativity and protective factors with depression, anxiety, non-suicidal self-injury and suicidal tendency in trans populations: A systematic review. Frontiers in Psychiatry.

[CR19] Israel, G. D. (1992). Determining sample size. *University of Florida: IFAS extension, PEOD-6.* https://www.psycholosphere.com/Determining%20sample%20size%20by%20Glen%20Israel.pdf

[CR20] Johns MM, Beltran O, Armstrong HL, Jayne PE, Barrios LC (2018). Protective factors among transgender and gender variant youth: A systematic review by socioecological level. Journal of Primary Prevention.

[CR21] King WM, Gamarel KE (2021). A scoping review examining social and legal gender affirmation and health among transgender populations. Transgender Health.

[CR22] Kung, H. H. (2020). 高中同志教育教材分析-以公民與社會科為例 [An analysis of high school LGBT education textbooks, taking civil and social sciences as an example]. (Master’s thesis). https://ndltd.ncl.edu.tw/cgi-bin/gs32/gsweb.cgi/login?o=dnclcdr&s=id=%22108NCUE5636010%22.&searchmode=basic

[CR23] Lerner UE, Lee JJ (2021). Transgender and gender diverse (TGD) Asian Americans in the United States: Experiences of violence, discrimination, and family support. Journal of Interpersonal Violence.

[CR24] Liu, C. H. (2022, November 11). 溫泉會館驚聲尖叫!男夾「卵蛋」混入女湯 堅稱「心理是女性」[Screaming in the hot spring! The male had scrotums went into women SPA and insisted his gender identity was female]. *Liberty Times Net*. https://news.ltn.com.tw/news/society/breakingnews/4119800?fbclid=IwAR2OyfhWUUAtZYe6mIDiHXq7Urw1yC-PylB-1Z0hjhiwQ7pmu8eYU0NRZg

[CR25] Long, B. A. (2022, Jan 19). 台灣史上第1次 112全中運開放跨性別選手參賽[2023 National High School Games to allow trans girls to compete in girls’ sporting events]. *CNA.* https://www.cna.com.tw/news/aspt/202201190391.aspx

[CR26] Manning, S. (2019, October 5). Girls are skipping school to avoid sharing gender neutral toilets with boys after being left to feel unsafe and ashamed. *Daily Mail*. https://www.dailymail.co.uk/news/article-7542005/Girls-skipping-school-avoid-sharing-gender-neutral-toilets-boys.html.

[CR27] Ministry of Interior. (2008). 戶政機關受理性別變更登記之認定要件[The requirements for the household registration agency to accept the registration of gender change]. https://gazette.nat.gov.tw/EG_FileManager/eguploadpub/eg014210/ch02/type2/gov10/num2/oEg.pdf

[CR28] Ministry of Health and Welfare. (2013). 12月9日性別變更登記認定要件研商會議之說明[Gender Change Registration Recognition Requirement Study and Consultation Meeting]. https://www.mohw.gov.tw/cp-3219-22594-1.html

[CR29] Morgenroth T, Sendén MG, Lindqvist A, Renström EA, Ryan MK, Morton TA (2021). Defending the sex/gender binary: The role of gender identification and need for closure. Social Psychological and Personality Science.

[CR30] Newman, J. (2019, March 15). Transgender woman, 18, sexually assaulted girl, 10, in Morrison's female toilets- just weeks after using mobile to peep on another girl in Asda loos. *Dail Mail*. https://www.dailymail.co.uk/news/article-6814599/Transgender-woman-18-sexually-assaulted-girl-10-Morrisons-female-toilets.html

[CR31] Restar A, Jin H, Breslow A, Reisner SL, Mimiaga M, Cahill S, Hughto JMW (2020). Legal gender marker and name change is associated with lower negative emotional response to gender-based mistreatment and improve mental health outcomes among trans populations. SSM-Population Health.

[CR32] Shaw, D. (2020, June 30). Male elected to female leadership seat created to ensure female participation in politics continue reading male elected to female leadership seat created to ensure female participation in politics. *Women Are Human*. https://www.womenarehuman.com/man-elected-to-female-leadership-seat-that-was-created-to-ensure-female-participation-in-politics/

[CR33] Taipei High Administrative Court. (2021). 臺北高等行政法院109年度訴字第275號原告江君 (代稱) 與被告桃園市大溪區戶政事務所間戶政事件新聞稿 [Press Release of the Household Registration-Plaintiff Jiang (assumed name) and the Defendant, the Household Registration Office of Daxi District, Taoyuan City]. https://www.judicial.gov.tw/tw/cp-1888-501330-16653-1.html

[CR34] United Nations. (2023). *The struggle of trans and gender-diverse persons.* https://www.ohchr.org/en/special-procedures/ie-sexual-orientation-and-gender-identity/struggle-trans-and-gender-diverse-persons

[CR35] Walker, P. (2023, February 16). Trans violent offenders banned from women’s prisons in England and Wales. *The Guardian.* https://www.theguardian.com/society/2023/feb/26/transgender-women-male-genitalia-banned-from-womens-prisons

[CR36] Wang, J. Q. (2021, November 2). 女全裸泡湯驚遇男員工 知名溫泉酒店道歉[A well-known hotel apologized for male employee entering the spa for unclothed women]. *Chinatimes* https://www.chinatimes.com/realtimenews/20211102002570-260405?chdtv

[CR37] Wang YC, Chang SR, Miao NF (2021). Suicide attempts among Taiwanese lesbian, gay, bisexual, and transgender adults during the 2018 Taiwan referendum on same-sex issues. Journal of Nursing Scholarship.

[CR38] World Health Organization. (2022a). *Gender incongruence and transgender health in the ICD*. https://www.who.int/standards/classifications/frequently-asked-questions/gender-incongruence-and-transgender-health-in-the-icd

[CR39] World Health Organization. (2022b). *Gender and health*. https://www.who.int/health-topics/gender#tab=tab_1

[CR40] Zhang, L. (2019, June 18). Taiwan: Same-sex marriage law enters into effect. *Library of Congress.* https://www.loc.gov/item/global-legal-monitor/2019-06-18/Taiwan-same-sex-marriage-law-enters-into-effect/

[CR41] Zimman L (2019). Trans self-identification and the language of neoliberal selfhood: Agency, power, and the limits of monologic discourse. International Journal of the Sociology of Language.

